# A tuberculosis outbreak in a psychiatric hospital: Kanagawa, Japan, 2012

**DOI:** 10.1017/S0950268819002206

**Published:** 2020-01-14

**Authors:** M. Tasaka, E. Koeda, C. Takahashi, M. Ota

**Affiliations:** 1 Kanagawa Prefectural Institute of Public Health, Kanagawa, Japan; 2 Kanagawa Prefectural Kamakura Public Health and Welfare Center, Misaki Branch, Kanagawa, Japan; 3 Research Institute of Tuberculosis, Kiyose City, Tokyo, Japan

**Keywords:** Contact investigation, disease outbreak, epidemiology, tuberculosis

## Abstract

In January 2012, an inpatient in a ward of a psychiatric hospital with nearly 300 beds in Kanagawa, Japan, was diagnosed with sputum smear-positive pulmonary tuberculosis (TB). Here we characterise the TB outbreak cases and identify the population at risk. TB was diagnosed when a person tested bacteriologically positive for TB or was determined to have TB by a physician. A latent TB infection (LTBI) case was defined as a person tested positive by interferon-gamma release assay (IGRA). A total of 125 contacts were screened via IGRA and chest X-ray. In all, 15 TB and 15 LTBI cases were found by the end of October 2012, and thereafter no additional TB case was found. Of the 15 TB cases, eight were culture-positive and all the isolates had identical variable number tandem repeat patterns. Twenty-four of the 56 (42.9%, 95% confidence interval (CI) 29.7–56.8) inpatients in the ward had either TB or LTBI with a relative risk of 8.6 (95% CI 1.2–59.3), compared to the staff members who did not work full-time in the ward (one of 20 (5.0%, 95% CI 0.0–24.9)). We recommend that psychiatric hospitals conduct periodic screening of staff members and inpatients for TB to prevent nosocomial TB outbreaks.

## Introduction

In Japan, the tuberculosis (TB) notification rate has declined in the past six decades from 698 per 100 000 population in 1951 to 17.7 per 100 000 population in 2013 [1]. However, about 8000 smear-positive TB cases are still reported every year, over 65% of which involve persons aged 65 years or older [2]. Elderly persons may be hospitalised in hospitals with geriatric wards, or sometimes in psychiatric hospitals because of dementia. TB outbreaks in these hospitals or nursing homes are not uncommon in Japan. According to the Ministry of Health, Labour and Welfare, there were 144 such outbreaks from 2006 through 2015 [3]. However, there have only been a few detailed studies on TB outbreaks or contact investigations related to psychiatric hospitals [4–6] or hospitals with geriatric wards [7], all of which were before 2006 when the interferon-gamma release assay (IGRA) [8,9] was not used in Japan.

The practice of contact investigations of TB contacts in Japan [10–12] is similar to that recommended elsewhere [13]; however, there are two differences. First, IGRA, rather than tuberculin skin testing (TST), is commonly used [14–16] to screen for latent TB infection (LTBI), because IGRA is more specific and can avoid interference caused by Bacillus Calmette–Guérin (BCG) vaccination, the rate of which is quite high (90%–95%) in the country [17]. Another advantage of IGRA over TST is that it only requires one patient visit for a test rather than two. Second, the use of a chest X-ray is recommended if there is a delay of more than 3 months between the onset of symptoms of a sputum smear-positive case and the diagnosis of TB [18].

In January 2012, an inpatient in his late 60s (patient A) hospitalised in ward Z of a psychiatric hospital with 300 beds in Kanagawa (near Tokyo), Japan, was diagnosed with sputum smear-positive pulmonary TB. Ward Z was a locked ward in which the inpatients were confined; however, they were free to move around inside the ward during the daytime. In March that year, two more inpatients in the same ward developed smear-negative TB with cough, and an administrative staff member who did not work in the ward developed smear-positive TB. The local public health office was notified about the patients in March, and, suspecting a TB outbreak, it started an investigation.

This study aims to characterise the outbreak cases, identify persons at risk and make recommendations to prevent similar outbreaks, particularly in psychiatric hospitals.

## Methods

A TB case was defined as one in which a patient had (1) bacteriologically positive TB in a sputum sample determined by smear microscopy, culture or nucleic acid amplification, or (2) a patient was determined to have TB by a physician via a chest X-ray, histological or pathological tests and who also had an epidemiologic link with one of the smear-positive outbreak-related cases. However, those culture-positive cases in which the 12-loci variable number tandem repeat (VNTR) [19 20] test did not match with patient A were excluded from the outbreak cases. An LTBI case was defined as one in which a patient had contact with smear-positive outbreak-related TB case(s) and tested positive in an IGRA test but had no chest X-ray findings suggestive of TB. A case of TB infection was defined as either a TB case, regardless of the availability of IGRA, or an LTBI case.

This is a retrospective cohort study. The cohort consisted of the staff and former staff members and the inpatients and former inpatients who worked or were hospitalised in ward Z of the hospital from October 2011 through December 2012 for at least 1 day and were considered to have had contact with patient A or other smear-positive outbreak TB patients. These included the nurses and assistants who worked full-time in ward Z (staff in ward Z), the nurses and assistants who had worked full-time in ward Z but had stopped their work when patient A was still in the ward (former staff in ward Z), and other staff members who did not work full-time in ward Z (non-ward Z staff), including medical doctors and occupational therapists. The proportions of the staff members and inpatients who developed TB disease or who were infected with TB were analysed using five categories: the staff and former staff members in ward Z, the inpatients and former inpatients in ward Z, and non-ward Z staff. Relative risks were calculated using the non-ward Z staff members as a reference.

To determine the background level of IGRA positivity among inpatients as a sort of ‘control’, inpatients in another ward of the hospital were also tested with IGRA.

The infectious period of patient A was determined to be from October 2011 through January 2012 when the patient was diagnosed with TB based on the respiratory signs and symptoms according to his chart. Since patient A's infectious period was more than three months, screening with both chest X-rays and IGRA was conducted for almost all the contacts. Screening with IGRA was conducted 3 months after the presumed last contact with a smear-positive patient, and it was repeated after several presumably secondary smear-positive patients were found. Chest X-ray screening was also repeated every six months until the end of 2014, when the outbreak was declared contained.

All the IGRA-positive contacts were screened by chest X-ray and those who had abnormal findings were referred to a chest physician who was familiar with TB for further investigation. The LTBI patients were prescribed six to nine months of isoniazid.

Statistical tests, including calculations of 95% confidence intervals (CIs), were carried out with R (The R Foundation, Vienna, Austria). Fisher's exact test was employed for comparison of proportions. The Cochran–Armitage test was conducted for the trend of proportions among the groups. A *P*-value of less than 0.05 was considered statistically significant.

## Results

A total of 125 individuals were enrolled as contacts for screening. Of these, all underwent chest X-ray examination and 120 (96.0%) had the IGRA test (Table 1). Altogether, 15 cases of TB disease (one staff member (an occupational therapist) and 14 inpatients) and an additional 15 LTBI cases (two staff members, 10 inpatients and three former inpatients in ward Z) were found by the end of October 2012, after which no TB patients were detected up to December 2014. Only three patients, patient A and the two inpatients diagnosed in March 2012 had respiratory symptoms. Overall, 24% (30/125) of those screened were infected with TB.

**Table 1.** Characteristics of the tuberculosis contacts in a psychiatric hospital, Japan, 2012

	Number	Age (median, range)	Female (%)	IGRA test done	Chest X-ray taken
Staff members					
Ward Z	31	52 (31–72)	26 (84)	31	31
Non-ward Z	20	39 (23–52)	13 (67)	19	20
Former staff members in ward Z	11	36.5 (28–53)	10 (91)	11	11
Inpatients in ward Z	56	54 (18–95)	30 (58)	52	56
Former inpatients in ward Z^a^	7	n/a	n/a	7	7
Total	125	–	–	120	125

^a^Detailed data on former patients in ward Z were not available.

One administrative staff member developed sputum smear-positive TB in March 2012; however, the VNTR pattern did not match with the other patients’ and that patient was excluded from the outbreak cases. Five colleagues of the staff member with TB underwent IGRA and chest X-ray examination, and one tested positive for IGRA and was put on LTBI treatment (not included in the outbreak-related cases). None developed TB disease during the study period.

To determine the background level of IGRA positivity, an additional 70 inpatients in another ward were also tested with IGRA and none was positive.

**Fig. 1.**
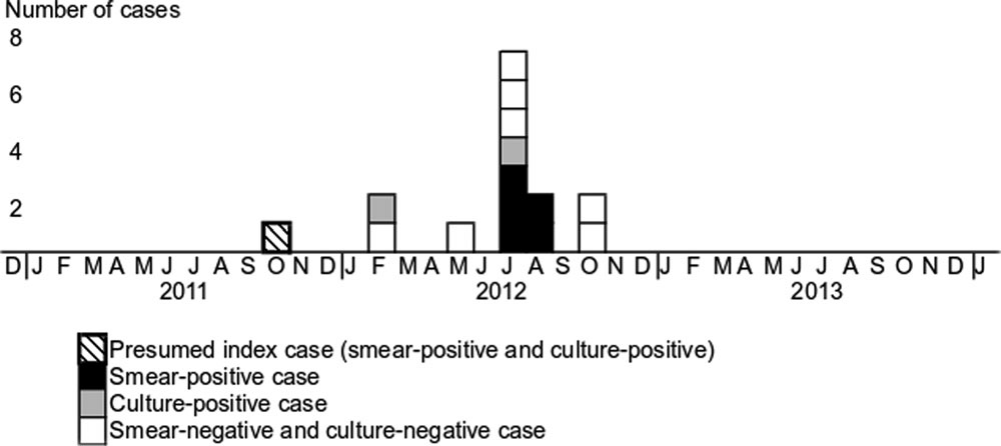
Epidemic curve of tuberculosis cases in a psychiatric hospital, Japan, 2012. The onset of illness or timing of diagnosis, if asymptomatic, is shown in the graph.

The epidemic curve is shown in Figure 1. Most TB patients were found in July 2012 after chest X-ray and IGRA screening was conducted in June 2012. Three smear-positive patients were found in July and two in August 2012. No TB patient was found from 2009 through 2011 (before the current outbreak), but one TB patient was found in 2008 in a different ward of the hospital. At the time, a contact investigation was conducted and no other TB patient was found. Table 2 shows a summary of the characteristics of the patients with TB disease and LTBI. The proportion of TB disease among those infected with TB was significantly higher as age increased (*P* < 0.03). Table 3 shows the risk of developing TB and contracting TB infection among various categories of staff members and inpatients. The highest risk of TB infection was seen in the inpatients and former inpatients of ward Z (both 42.9%, 95% CIs 29.7–56.8 and 9.8–81.6, respectively), followed by the staff members of ward Z (6.5%, 95% CI 0.8–21.4) and non-ward Z staff members (5.0%, 95% CI 0.0–24.9). The inpatients and former inpatients in ward Z were 8.6 times (95% CIs 1.2–59.3 and 1.1–69.5, respectively) more likely to be infected with TB than the non-ward Z staff members.

**Table 2.** Characteristics of the cases with tuberculosis (TB) and latent TB infection (LTBI) in a psychiatric hospital, Japan, 2012

	TB disease	LTBI	%TB disease (95% CI)	Total
Number	15	15	50.0 (31.2–68.7)	30
Sex				
Male	6	7	46.2 (19.2–74.9)	13
Female	9	8	52.9 (27.8–77.0)	17
Age				
0–34	1	0	100 (2.5–100)^a^	1
35–44	0	2	0 (0–84.2)^a^	2
45–54	0	4	0 (0–60.2)^a^	4
55–64	4	5	44.4 (13.7–78.8)^a^	9
65–74	4	3	57.1 (18.4–90.1)^a^	7
75+	6	1	85.7 (42.1–99.6)^a^	7
Site of TB				
Pulmonary	12	–	–	–
Pleural	3	–	–	–
Bacteriological confirmation				
Smear- and culture-positive	6	–	–	–
Smear-negative and culture-positive	2	–	–	–
Smear-negative and culture-negative	7	–	–	–

LTBI, latent tuberculosis infection; TB, tuberculosis.

^a^The proportion of TB disease among TB disease and LTBI statistically significantly (*P* < 0.03) increased as the age of the group increased.

**Table 3.** Risk of contracting tuberculosis disease and infection among staff members and inpatients in a psychiatric hospital, Japan, 2012

	TB disease		TB disease + LTBI		Total
	*n* (%, 95% CI)	RR (95% CI)	*n* (%, 95% CI)	RR (95% CI)	
Inpatients in ward Z	14 (25.0, 14.4–38.4)	5.0 (0.7–35.6)	24 (42.9, 29.7–56.8)	8.6 (1.2–59.3)^a^	56
Former inpatients in ward Z	0 (0.0, 0.0–41.0)	0 (–)	3 (42.9, 9.8–81.6)	8.6 (1.1–69.5)^a^	7
Staff in ward Z	0 (0, 0.0–11.2)	0 (–)	2 (6.5, 0.8–21.4)	1.3 (0.1–13.3)	31
Former staff in ward Z	0 (0, 0.0–28.5)	0 (–)	0 (0, 0.0–28.5)	0 (–)	11
Non-ward Z staff^b^	1 (5.0, 0.0–24.9)	ref.	1 (5.0, 0.0–24.9)	ref.	20
Total	15 (12.0, 6.9–19.0)	–	30 (24.0, 16.8–32.5)	–	125

CI, confidence interval; ref., reference; RR, relative risk; TB, tuberculosis.

a Relative risks are statistically significant.

b Including medical doctors and occupational therapists who had minimal contacts with the index patient (patient A).

Of the 15 cases of TB disease, cultures from eight cases, including that of the occupational therapist, yielded TB bacilli, and VNTR analysis revealed that the strains of all eight culture-positive cases were identical.

## Discussion

We conducted an investigation of a TB outbreak in a psychiatric hospital in Japan with a large number of the staff members and inpatients tested using IGRA. The inpatients and former inpatients who were hospitalised in the same ward as patient A were 8.6 times more likely to be infected with TB than the non-ward Z staff members who had minimal contact with patient A, whereas the risk of TB infection of the staff members in ward Z was not different from that of the non-ward Z staff members. We also found that the proportions of those who developed TB disease among those infected with TB were higher as age increased. Since the VNTR patterns of all the culture-positive cases were identical, the event was considered to be an outbreak involving one strain of TB bacilli. The outbreak was contained in October 2012, after which no additional cases of TB disease were detected.

The reason why the risk of TB infection among the inpatients and former inpatients in ward Z was higher than for the staff members of the same ward or non-ward Z staff members was that the cumulative time spent with patient A was longer for the fellow inpatients than for the staff members [5 6 21–23]. Since TB is transmitted by air, the index patient's interactions with other patients within a short distance were not an important point. Rather, the cumulative time they spend in the same air space with the index patient was more important [24] in addition to overcrowding and poor ventilation. When the outbreak occurred, particularly the patient A's infections period (October 2011 to January 2012), probably the area was poorly ventilated to keep the ward warm, thus increasing TB transmission. Elderly individuals are more likely to develop TB disease once they are infected with TB, particularly after the age of 55 years, because of underlying diseases such as diabetes mellitus [25], tobacco use [26] and the waning of specific immunity against TB bacilli [27]. This was theorised long ago based on indirect evidence [28]; however, to the best of our knowledge, neither supporting nor contradictory evidence has been reported. In this regard, our findings may offer direct evidence for the theory. After October 2012, no additional patients developed TB, probably because LTBI treatment for the IGRA-positive inpatients and staff members was effective in preventing them from developing TB disease. The authors would like to emphasise that this is truly great progress made via the introduction of IGRAs because it was not possible to specifically find LTBI cases and thus to contain TB outbreaks early and effectively before the introduction of IGRAs in a country with high-coverage BCG vaccination [6 17]. Since all the secondary smear-positive inpatients (i.e. other than patient A) developed symptoms or, if asymptomatic, were diagnosed in July and August, it is likely that patient A, who developed symptoms in December the previous year (2011), was the source case. Since the administrative staff member who had smear-positive TB with a different VNTR pattern from the others did not have contact with the inpatients in ward Z, it is unlikely that this staff member spread TB to the inpatients in the ward. Thus, we are confident that the administrative staff member's development of TB was a separate event from the outbreak.

There were a few reports on TB outbreaks involving psychiatric hospitals in Japan in the late 1990s and early 2000s [3–5]. The situations were similar to the current one as the index cases were inpatients that presumably spread TB to multiple other inpatients and a few or no staff members of the same ward who developed TB disease. The strains of TB bacilli were confirmed to be identical for each outbreak. However, at that time no IGRAs were available and the extent of TB infection among the inpatients and staff members could not be assessed. In other parts of the world there have been similar findings. In a hospital for mentally handicapped patients in Havana, Cuba, 14 inpatients and a health care worker developed TB disease from 1995 through 1998 [21], of whom 12, including the health care worker, had the same strain. The index patient was presumed to be an inpatient who developed the disease in early 1995. However, no findings were presented on the magnitude of the TB infection among the inpatients and health care workers. In a psychiatric health care facility in Taiwan, 17 inpatients, but no health care worker, developed TB disease from 2011 through 2015 [22]. An inpatient diagnosed in 2011, the only one who was sputum smear-positive, was presumed to be the index TB patient. Ten culture-positive patients, including the index patient, had identical VNTR patterns. No findings were presented on the magnitude of TB infection among the inpatients and health care workers. In a long-term care facility for mentally ill persons in Puerto Rico, seven residents developed TB disease in the period from 2010 to 2012 [23]. TST revealed evidence of LTBI in 26 (81%) of the 32 residents and seven (5%) of the 155 non-resident contacts (facility employees and residents’ family members).

Our study has some strengths. First, since we frequently screened the inpatients of ward Z by chest X-ray, the time sequence in which the secondary patients developed TB was accurate and they were considered to be incident, not prevalent, cases. Second, since we screened almost all the staff members and inpatients who we thought had contact with patient A and the other outbreak-related sputum smear-positive patients, particularly with IGRA, we were able to assess the magnitude of TB infection as well as TB disease, and the findings we present here are not considered selection biased. Third, since we established a sort of background level of zero IGRA positivity by screening the inpatients of another ward, we were able to show that the other wards were not affected by the outbreak.

One of the limitations was that IGRAs do not date the occurrence of infection and some of the 15 IGRA-positive contacts might possibly have been infected with TB long before or from sources other than the index patient. However, the findings that the risk of TB infection was parallel that of TB disease among the five categories of contacts (Table 3), most patients with TB disease were infected with the same strain of TB bacilli, and the IGRA-positivity among the inpatients in another ward was zero suggest that spread of TB infection in the psychiatric hospital was relatively rare and that most, if not all, IGRA-positive contacts had acquired TB infection recently, most probably from patient A. Second, we do not know whether there was a link between the TB patient in 2008 and patient A because no sample was kept from the 2008 patient and we could not conduct VNTR analysis. However, considering that the 2008 patient was hospitalised in a different ward and no other TB patient was found after the contact investigation at that time, it is unlikely that there was a link. Third, this study was based on observations in a single psychiatric hospital. However, we believe it might be possible to extrapolate the results to long-term care wards in psychiatric hospitals or hospitals with geriatric wards of other countries with a medium burden of TB similar to Japan.

We can make a few recommendations to psychiatric hospitals with long-term care wards in countries with a medium- or high-burden of TB based on our and other TB outbreak investigations. To minimise the delay in detection of TB cases, we recommend that the hospitals conduct periodic screening of staff members and inpatients for TB. Second, if anyone has a persistent, unexplained cough, appropriate measures, including sputum examinations and chest X-rays, if indicated, should be taken. In a country with high BCG coverage, it is recommended to national TB programmes that IGRAs be used rather than TST to identify LTBI cases in TB contact and outbreak investigations. In addition, persons in psychiatric hospitals with LTBI who have had close contact with a sputum smear-positive TB patient should be treated to the degree possible to contain the outbreaks in these institutions.
